# *Staphylococcus aureus* colonization and non-influenza respiratory viruses: Interactions and synergism mechanisms

**DOI:** 10.1080/21505594.2018.1504561

**Published:** 2018-08-26

**Authors:** M. Fedy Morgene, Elisabeth Botelho-Nevers, Florence Grattard, Sylvie Pillet, Philippe Berthelot, Bruno Pozzetto, Paul O. Verhoeven

**Affiliations:** a GIMAP EA 3064 (Groupe Immunité des Muqueuses et Agents Pathogènes), University of Lyon, Saint-Etienne, France; b Infectious Diseases Department, University Hospital of Saint-Etienne, Saint-Etienne, France; c Laboratory of Infectious Agents and Hygiene, University Hospital of Saint-Etienne, Saint-Etienne, France

**Keywords:** *Staphylococcus aureus*, non-influenza respiratory viruses, human rhinovirus, respiratory syncytial virus, virus-bacterium interaction, nasal carriage

## Abstract

Viral infections of the respiratory tract can be complicated by bacterial superinfection, resulting in a significantly longer duration of illness and even a fatal outcome. In this review, we focused on interactions between *S. aureus* and non-influenza viruses. Clinical data evidenced that rhinovirus infection may increase the *S. aureus* carriage load in humans and its spread. In children, respiratory syncytial virus infection is associated with *S. aureus* carriage. The mechanisms by which some non-influenza respiratory viruses predispose host cells to *S. aureus* superinfection can be summarized in three categories: *i)* modifying expression levels of cellular patterns involved in *S. aureus* adhesion and/or internalization, *ii)* inducing *S. aureus* invasion of epithelial cells due to the disruption of tight junctions, and *iii)* decreasing *S. aureus* clearance by altering the immune response. The comprehension of pathways involved in *S. aureus*-respiratory virus interactions may help developing new strategies of preventive and curative therapy.

## Introduction

The development of upper and lower respiratory tract infections is determined by the interaction between one micro-organism and the host immune response. More recently, the interaction between the resident microbiota and incoming pathogens may also participate in the development of respiratory tract infections.

It has been postulated for a long time that viral infection of the respiratory tract indirectly predispose to bacterial superinfection by disruption of the respiratory mucosal epithelium [1], or passively through anatomical and mechanical changes like Eustachian tube dysfunction [2], ostiomeatal obstruction and reduced mucocilliary clearance [3]. Over the last decades, there was an increasing interest to investigate the contribution of bacterial colonization in the outcome of viral respiratory tract infections. The human respiratory tract is known to be the reservoir of diverse commensals and potential pathogens including mainly *Staphylococcus aureus, Streptococcus pneumoniae* and *Haemophilus influenzae* that compose a significant part of the respiratory tract microbiota [4].

The role of interactions between viruses and bacteria in the pathogenesis of respiratory infections have been extensively studied in the literature and notably those between influenza viruses and *S. aureus* or *S. pneumoniae* [5–10]. However, interactions between *S. aureus* and non-influenza respiratory viruses were not reviewed recently.

The aim of this report is to describe the current knowledge on possible interactions between *S. aureus* and non-influenza viral pathogens in the respiratory tract, with a focus on the mechanisms by which these interactions are potentially mediated.

## Impact of viral infections in the respiratory tract on *staphylococcus aureus* colonization

*S. aureus* is a commensal bacterium of the skin and mucosa, colonizing 15 to 36% of the whole population [11,12]. The main reservoir of *S. aureus* carriage in humans remains the nose [12], but other sites of carriage have been reported, such as the skin [11], pharynx [13,14], vagina [15], and rectum [16]. *S. aureus* is however a Janus-faced bacterium and beyond its commensal status, it is also a life-threatening pathogen. It is in fact considered to be one of the leading causes of nosocomial and community-acquired bacterial infections [17]. In addition to be the most common cause of bacteremia, with a 25% mortality rate despite appropriate treatment [18], it is also known as an etiological agent of other deep-seated infections including osteomyelitis, septic arthritis, endocarditis and device-related infections [17,19].

*S. aureus* is also an important pathogen in lung infection, mostly implicated in hospital-acquired pneumonia [17]. *S. aureus* expresses a wide repertoire of surface proteins that recognize cellular adhesive molecules and it is therefore able to adhere to and internalize into lung epithelial cells, which protects the bacteria from the host immune system and facilitate chronic infection [20]. *In vitro* experiments have demonstrated that the activation of NF-κB (nuclear factor kappa-light-chain-enhancer of activated B cells) signaling pathway in infected pulmonary epithelial cells results in inflammation enhancement via IL-8 expression; furthermore, intracellular *S. aureus* can lead, after an initial lag period, to the apoptosis of these cells. [21,22] In necrotizing pneumonia, the key virulence factors of *S. aureus* associated with the apoptosis of lung cells were shown to be pore-forming toxins, namely Panton-Valentine leukocidin (PVL) and alpha-hemolysin [23,24].

Besides, *S. aureus* is frequently involved in secondary bacterial pneumonia occurring during seasonal influenza outbreaks [8]. During the 2009 A/H1N1 influenza pandemic, bacterial co-infections complicated up to one-third of influenza cases in the United States in which *S. aureus* was the most common pathogen, accounting for 27% of the cases in both critically ill children and adults [25,26]. *S. aureus* co-infection was associated with significantly higher morbidity and mortality [26]. To date, the clinical association between the healthy carriage of *S. aureus* and the secondary staphylococcal pneumonia is still unclear. However, recent evidence from *in vitro* and *in vivo* tests showed that host physiologic changes induced by influenza virus can lead to the transition from asymptomatic colonization to invasive disease [27]. In addition to influenza viruses, *S. aureus* nasopharyngeal carriage has been found to be associated with some other respiratory viruses like rhinovirus and respiratory syncytial virus (RSV).

### Staphylococcus aureus *and rhinovirus*

Rhinovirus is the second respiratory virus that has been most frequently reported to interact with *S. aureus*. Several studies showed that natural or experimental rhinovirus infection in *S. aureus* nasal carriers leads to increased *S. aureus* airborne dispersal, especially when sneezing is a part of the syndrome [28–33]. These studies emphasized that rhinovirus infection may facilitate the spreading of *S. aureus* from staphylococcal carriers to their environment and the transmission of the bacterium between humans. A recent study showed that rhinovirus infection is associated with changes in the upper respiratory tract microbiota [34]. In this study, healthy adults who were experimentally infected by rhinovirus showed increase in the relative abundance of *H. parainfluenzae, Neisseria subflava* and *S. aureus*, and returned to their baseline level after the infection was cleared [34]. It has been also shown that experimental rhinovirus infection significantly increases *S. aureus* nasal load by 39% compared to baseline bacterial load [30]. These findings suggest that changes in the composition of respiratory microbiota following rhinovirus infection may play a role in the development of bacterial superinfection. However, the role of rhinovirus infection on the onset or increase of staphylococcal carriage in human remains poorly studied.

### Staphylococcus aureus *and respiratory syncytial virus*

A prospective microbiological analysis showed that 40% of children with severe RSV bronchiolitis had a bacterial co-infection in their lower airways and were at increased risk for bacterial pneumonia [35]. Bacterial co-infection in children with RSV bronchiolitis seems to increase inflammatory markers, abnormal radiologic patterns and hospital stay [36,37]. In young infants with RSV bronchiolitis, *S. aureus, H. influenzae, M. catarrhalis* and *S. pneumoniae* are the most common pathogens colonizing the nasopharynx and the lower airways [35,36].

A recent prospective study investigated the differences in the nasopharyngeal microbiome during acute respiratory tract infections due to human rhinovirus or RSV in 135 infants aged less than 6 months [38]. By contrast to previous studies, *S. aureus* was not found among the most abundant bacteria. However, the difference of *S. aureus* abundance was significantly higher in RSV than in rhinovirus-infected infants [38]. Another prospective study investigated the nasopharyngeal microbiota in young infants with RSV infections by 16S-RNA sequencing; the authors found that RSV infection was positively associated with *H. influenzae* and *S. pneumoniae*, but negatively associated with *S. aureus* nasopharyngeal colonization [39]. In another study concerning nasopharyngeal aspirates from children with RSV infection aged between 6 months and 2 years, *S. aureus* was shown to colonize 77% of RSV-infected patients with a positive association between RSV and *S. aureus* nasopharyngeal carriage [40]. The conflicting data from the two latter studies may be explained by differences in target populations, sampling procedures and/or microbiological methods.

To date, most of the studies dedicated to the impact of RSV infection on the bacterial colonization of the upper respiratory airways identified a synergistic interaction between RSV and *S. pneumoniae* [41,42]. Nevertheless, nasopharyngeal colonization with *S. aureus* is clearly more than a passive phenomenon during RSV infection and further studies are needed to elucidate the interactions between these 2 pathogens.

There is no doubt that the impact of infection with respiratory non-influenza viruses on *S. aureus* colonization is important and should not be neglected as this may worsen the disease outcome and even be fatal in some cases [43]. The recent technical breakthrough of molecular diagnostic will be of precious help to investigate the changes in nasopharyngeal microbiota composition and notably in staphylococcal carriage during non-influenza viral infections of the respiratory tract.

## Mechanisms involved in interactions between *staphylococcus aureus* and non-influenza respiratory viruses

The high incidence of staphylococcal superinfection during pandemic and seasonal influenza and the important mortality risk associated to this condition promoted the in-depth investigation of the molecular and immunologic mechanisms that are involved in the bidirectional synergism between both pathogens. Influenza virus is known to promote staphylococcal superinfection by alteration of the host immune system via increased production of pro-inflammatory cytokines and interferons (IFNs), impairment of phagocytic cell functions and suppression of type 17 immunity [9,10]. In addition, influenza virus has been shown to promote *S. aureus* adhesion and internalization within non-professional phagocytic cells through at least two distinct mechanisms: binding of bacteria to the membrane-associated hemagglutinin of influenza-infected cells, and binding of bacteria to free virions, followed by internalization of virus-coated bacteria into non-infected cells [44]. Besides, *S. aureus* co-infection promotes influenza virus replication and pathogenicity; indeed, extracellular bacteria secrete staphylokinase that facilitates the binding of influenza virus to the host cells, whereas intracellular *S. aureus* inhibits influenza virus-induced type I IFN signaling through impaired signal transducers and activators of transcription (STAT1 and STAT2) dimerization [45,46]. While the mechanisms of interaction between *S. aureus* and influenza virus seem to be deeply understood, the interactions between *S. aureus* and other respiratory viruses were less investigated. Table 1 summarizes the main mechanisms and pathways potentially involved in the interactions between *S. aureus* and non-influenza respiratory viruses.

**Table 1.** Virulence factors involved in molecular mechanisms of interactions between *Staphylococcus aureus* and non-influenza respiratory viruses.

Virulence factors	Cellular target	Effects	Synergism	References
***Staphylococcus aureus***				
SEA/SEB	Unknown	Increase of IL-1β, IL-6 and IL-8 secretion	Increase of ICAM-1 expression Enhancement of rhinovirus replication	[53,83–85]
LTA	Unknown	Increase of IL-6, IL-12/IL-23 and IFN-γ secretion	Increase of susceptibility to coronavirus	[98]
**Human rhinovirus**				
Capsid	Membrane TLR2	NFκB activation Increase of IL-6, IL-8, INFβ and IFN-γ secretion	Increase of cFn and ICAM-1 expression Increase of *S. aureus* adhesion and internalization to/in host cells	[48,51–56]
ssRNA	Endosomal TLR7/8			
	Cytoplasmic RIG-I	Increase of IFN-β, IFN-γ, RANTES, IL-8, IP-10 and ENA78 secretion		
dsRNA	Cytoplasmic MDA5			
	Endosomal TLR3	RIG-I and MDA5 upregulation NFκB activation Increase of IL-6, IL-8, INF-β and IFN-γ secretion		
	Unknown	Rac1 activation NOX1 production Stimulation of ROS generation	Loss of ZO-1 tight junctions Induce *S. aureus* invasion of the epithelium	[78,81]
**Respiratory Syncytial Virus**				
Viral envelope	Membrane TLR2	NFκB activation Increase of pro-inflammatory cytokine secretion IRFs activation Increase of type I IFNs secretion	Alteration of neutrophil recruitment Inhibition of B cells response Decrease of *S. aureus* clearance	[87,90,93,94,99]
Viral F protein	Membrane TLR4			
ssRNA	Endosomal TLR3			
	Cytoplasmic RIG-I			
	Cytoplasmic Nod2			
dsRNA	Endosomal TLR7			
	Cytoplasmic MDA5			
Unknown	NKG2D	Increase of IFN-γ secretion		
Unknown	Unknown	Increase of Lewis blood group antigen expression	Increase of toxin-producing *S. aureus* binding to epithelial cells	[88,89,91]

### Staphylococcus aureus *and rhinovirus*

Rhinovirus, the most common cause of upper respiratory tract infections (URTI), primarily targets the nasal and nasopharyngeal epithelial cells [47]. The innate immune system recognizes rhinovirus by different Pattern Recognition Receptors (PRRs) including membrane and endosomal Toll-like Receptors (TLRs) and cytoplasmic inducible RNA helicases like retinoic acid-inducible gene-1 (RIG-I) or melanoma differentiation-associated protein 5 (MDA5) [48]. Rhinovirus infection promotes pro-inflammatory cytokines and IFN production mainly through the activation of NFκB [49,50].

Several potential mechanisms through which rhinovirus increases susceptibility to bacterial infection have been demonstrated *in vitro* in epithelial cells of the upper and lower airways. In 1981, Selinger, Reed and McLaren developed an *in vitro* model for studying bacterial adherence to virus-infected epithelial cells [51]. They reported that the adherence of *S. aureus* was significantly higher in rhinovirus-infected cells compared to uninfected cells. Only recently, various *in vitro* studies have shown that inflammation due to rhinovirus infection increased cellular patterns that facilitate the adhesion and internalization of *S. aureus* within host cells [52–56].

### Rhinovirus promotes cellular fibronectin expression

In primary human nasal epithelial cells, it has been demonstrated that rhinovirus infection up-regulates the expression of cellular fibronectin [54,56]. The up-regulation of fibronectin was observed at both transcriptional and translational levels and seems to be related to the rhinovirus-induced NFκB activation [56]. Fibronectin is known to mediate the adhesion and the internalization of *S. aureus* in the presence of epithelial cells [57]. The fibronectin-binding protein A or B (FnBPA/B) are *S. aureus* cell wall-anchored Microbial Surface Components Recognizing Adhesive Matrix Molecules (MSCRAMMs) that bind type I motif of fibronectin through a tandem beta-zipper interaction [58]. The α5β1 integrin expressed on host cellmembrane binds the RGD motif of fibronectin [59]. Fibronectin is thus acting as a bridge between *S. aureus* and the host cell, and the complex formed by FnBPA/B, fibronectin and α5β1 integrin leads to *S. aureus* internalization inside the epithelial cell [57]. Then the up-regulation of fibronectin expression in epithelial cells during rhinovirus infection could explain the increase of *S. aureus* epithelial cells adhesion [54,56].

### Rhinovirus promotes ICAM-1 expression

The intercellular adhesion molecule 1 (ICAM-1), which is the receptor of the major group of rhinoviruses [60], have been found to be overexpressed during rhinovirus infection [52,61]. Adhesion and internalization of *S. aureus* to epithelial cells is also mediated by ICAM-1 [52,54]. Both immortalized pneumocytes (A549 cell line) and primary human nasal epithelial cells have been found to release higher levels of IL-6 and IL-8 when infected by rhinovirus and subsequently overexpressed ICAM-1. The pro-inflammatory effect of IL-6 and IL-8 also induces an overexpression of ICAM-1 in the surrounding uninfected cells, which increases *S. aureus* uptake by the epithelial cells [52]. The up-regulation of ICAM-1 and pro-inflammatory cytokines in rhinovirus-infected epithelial cells seems to follow the NFκB pathway [61–63].

Interestingly, the extracellular adherence protein (EAP), an adhesin naturally secreted by *S. aureus*, was found to bind ICAM-1 [64]. EAP belongs to Secreted Expanded Repertoire Adhesive Molecules (SERAMs) and is involved in bacterial aggregation [65], adhesion and invasion of epithelial and fibroblastic cells [66–68], and in preventing neutrophil recruitment [64,69–72]. In addition to ICAM-1, EAP can also bind a large variety of extracellular matrix and plasma proteins including collagen, fibrinogen, fibronectin, vitronectin, laminin, thrombospondin and prothrombin [64,65,73–75]. Taken together, it can be hypothesized that the ligation of *S. aureus* to ICAM-1, whose expression is enhanced by rhinovirus infection, is mediated by EAP.

### Rhinovirus disrupts tight junctions

The mucosal barrier of the nasal cavity is the first site of exposure to inhaled respiratory pathogens and plays an important role in host defenses in terms of innate immunity. Its integrity is regulated in large part by tight junctions of epithelial cells [76], which is a complex of several internal and membrane proteins including occludin and zona occludens (ZO) proteins [77]. Rhinovirus infection disrupts the barrier function of the airway epithelium through the dissociation of ZO-1 and occludin from the tight junction complex [78]. Rhinovirus infection has been shown to induce oxidative stress in respiratory epithelial cells by generating reactive oxygen species (ROS) [79]. ROS generation is induced by double-strands of viral RNA (dsRNA) that appear transiently during rhinovirus replication, and is produced by NADPH oxidase [78]. In non-phagocytic cells, ROS act as a molecular switch to stimulate pro-inflammatory responses [80]. Nevertheless, ROS generation disrupts the barrier function of rhinovirus-infected cells [78]. Thus, a perturbation of the tight junction barrier function increases paracellular permeability, facilitates translocation of pathogens and their soluble products, and exposes basolateral receptors. It has been found that the infection of primary human airway epithelial cells with both major and minor groups of rhinovirus promoted the paracellular migration of *S. aureus* and its epithelium invasion [81]. The rhinovirus infection facilitates also the transmigration of other bacterial species like *H. influenzae* and *Pseudomonas aeruginosa* [81]. This provides insights into another mechanism by which rhinovirus can predispose the host to secondary bacterial infections.

### Staphylococcal enterotoxins a and b promote rhinovirus replication

Interactions between rhinovirus and *S. aureus* have been shown to be bi-directional: whereas rhinovirus promotes *S. aureus* adhesion and internalization within host cells, *S. aureus* enhances the rhinovirus replication [53]. *S. aureus* secretes several toxins including pore-forming toxins, toxic shock syndrome toxin and enterotoxins [82]. *In vitro*, staphylococcal enterotoxins A and B (SEA and SEB) were shown to enhance the rhinovirus replication in A549 epithelial cells in a dose-dependent manner; however, they were found able to enhance neither ICAM-1 expression nor Il-1β, IL-6 and IL-8 secretion [53]. In contrast, studies using other cellular models (normal human keratinocytes, coculture of human peripheral blood mononuclear cells and A549 cells, or primary nasal epithelial cell cultures) showed that these enterotoxins induced ICAM-1 expression or the secretion of pro-inflammatory cytokines [83–85]. Therefore, the mechanisms involved in the promotion of rhinovirus replication via staphylococcal enterotoxins need to be studied more in-depth.

**Figure 1.**
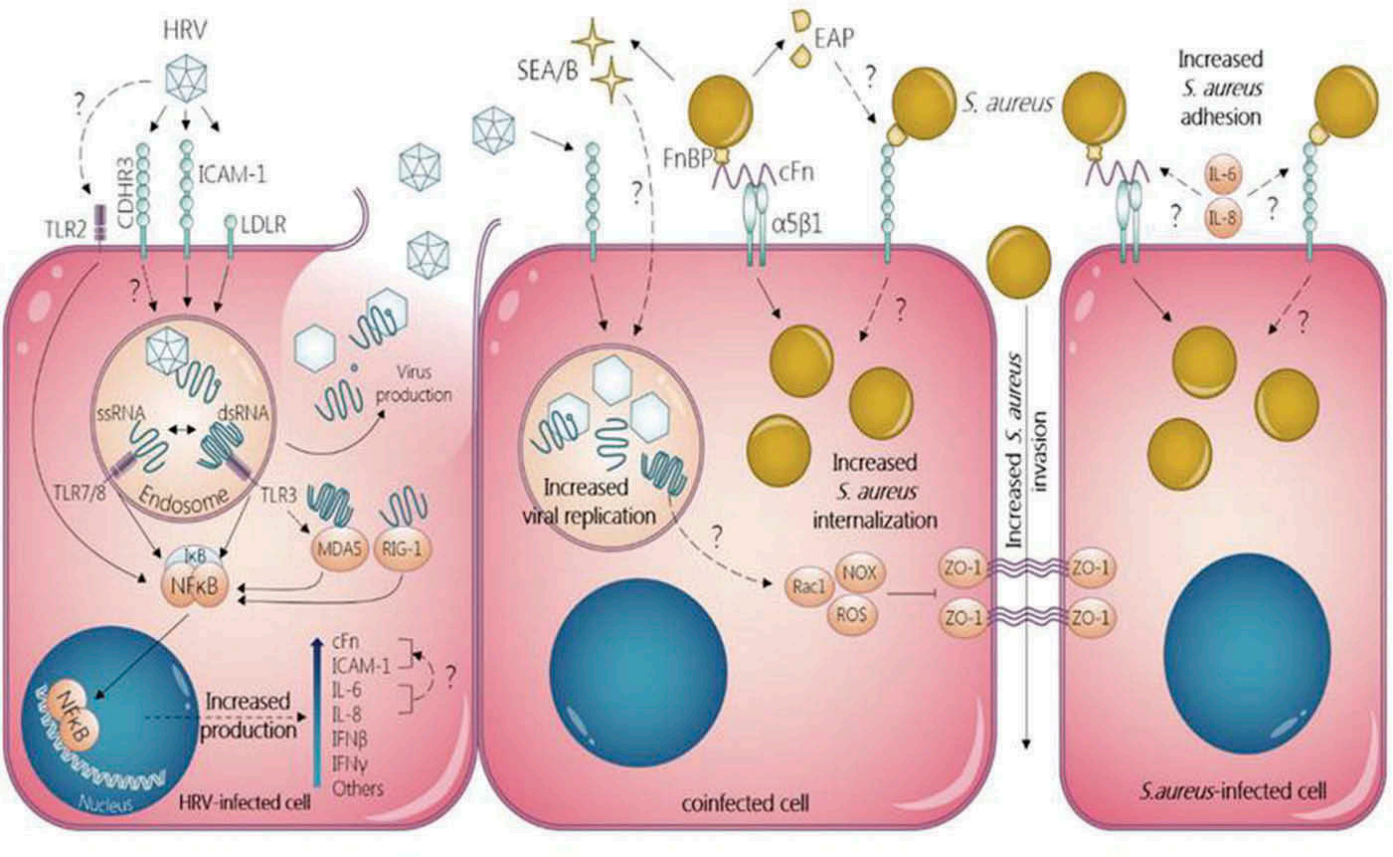
**Mechanisms of bi-directional synergism between *Staphylococcus aureus* and human rhinovirus in non-professional phagocytic epithelial cells**. *S. aureus* increases HRV replication via SEA and SEB. HRV increases adhesion and internalization of *S. aureus* in both HRV-infected and uninfected cells through increased release of IL-1β, IL-6 and IL-8 and subsequent increase of ICAM-1 and cFn expression via NFkB activation. HRV inhibits ZO-1 tight junctions favoring *S. aureus* invasion of the epithelium.

All these experimental data provide evidence of bi-directional synergism between rhinovirus and *S. aureus*. The different mechanisms described above are summarized in Figure 1. Nevertheless, the molecular pathways of these mechanisms remain poorly understood and deserve further investigation.

### Staphylococcus aureus *and respiratory syncytial virus*

RSV is a worldwide seasonal virus that affects mainly young children. RSV disease manifestations can vary from mild URTI to severe pneumonia or bronchiolitis, which can lead to hospitalization and serious complications like respiratory failure [86]. Despite the high incidence of bacterial and RSV co-infection in both children and adults, only few studies aimed to investigate the possible interactions between *S. aureus* and this virus [87–90]. RSV is supposed to exacerbate *S. aureus* co-infection via at least two mechanisms: enhancing the adhesion of *S. aureus* to the RSV-infected cells [88,89], and decreasing the bacterial clearance by altering the immune response to *S. aureus* [87,90].

### RSV infection enhances *staphylococcus aureus* adhesion

*In vitro* studies had shown that RSV-infected epithelial cells bind more *S. aureus* than uninfected cells [88,89]. Study of nasal washings in patients with respiratory virus diseases has shown that secretion of Lewis blood group antigens is associated with RSV infection [91]. In addition, epithelial cells (HEp2) expressing high concentrations of antigens of the Lewis blood group bound significantly more *S. aureus* than cells expressing low concentrations of these antigens [88,89]. While RSV infects about 50% of infants by the first year of life, Lewis antigen is expressed in secretions of nearly 90% of 3 month-aged infants [88,89]. This may explain the high susceptibility of RSV-infected infants to secondary staphylococcal infection, sometimes resulting in lethal issue [92]. Nevertheless, the molecular patterns involved in RSV-related increase of *S. aureus* adhesion to host cells remain unknown.

### RSV alters the immune response to *staphylococcus aureus*

Pro-inflammatory cytokines and type I IFNs are known to be secreted by RSV-infected epithelial cells after viral sensing by different PRRs, mainly through NFκB and Interferon Regulation Factors (IRFs) pathways [93]. In addition, IFN-γ (type II IFN) seems to play an important role in the immune response to RSV as it activates the T cell response [94]. It has been demonstrated that RSV infection of human adult or neonatal mononuclear leukocytes results in significant inhibition of the lymphoproliferative response to heat-killed *S. aureus* [87]. This inhibitory effect on the development of cell-mediated immune response could be due in part to the increased secretion of IFN-γ[87]. IFN-γ was shown to reduce RSV replication in epithelial cells and also to inhibit B cell responses, which may alter the humoral response to *S. aureus* infection and decrease the bacterial clearance [87,94].

Murine models had been used to demonstrate that, despite the increased number of inflammatory cells, RSV decreases the clearance of *S. aureus* and other pathogenic bacteria like *S. pneumoniae* and *P. aeruginosa* from the lungs of mice following secondary bacterial infection [90]. It was suggested that RSV alters neutrophil function via changes in inflammatory response and cytokine secretion in the lung. Further studies are needed to delineate the different immunological pathways that sustain the decrease of bacterial clearance and the increase of susceptibility to secondary infections by *S. aureus* following RSV infection.

### Staphylococcus aureus *and other respiratory tract viruses*

*S. aureus* interactions with rhinovirus or RSV have been actively, yet insufficiently, investigated. However, bacterial interactions with other respiratory viruses are poorly studied and only partial studies are available. For example, parainfluenza virus has been shown to enhance the ability of non-typeable *H. influenzae* and *S. pneumoniae* to adhere to human respiratory epithelial cells [95]. However, there is no information available about the interaction of these agents with *S. aureus* in humans. Only one *in vitro* study conducted on bovine embryonic lung cells investigated the effect of bovine parainfluenza virus infection on adherence of several bacterial agents, including *S. aureus*, and found no virus-specific effect on any of the tested bacteria [96]. Besides, normal human bronchial epithelial cells that were pre-incubated with *S. pneumoniae* resulted in an increased susceptibility to infection with human metapneumovirus. Nevertheless, this was not the case for cells pre-incubated with *H. influenzae, M. catarrhalis* or *S. aureus* [97]. On the contrary, synergistic effect between *S. aureus* and coronavirus has been demonstrated *in vivo* in a swine model. Lipoteichoic acid from *S. aureus* increased the susceptibility to coronavirus infection in pigs via increased secretion of pro-inflammatory cytokines IL-6, IL-12, IL-23 and IFN-γ[98]. To date, the lack of clinical and experimental data about the relationship between *S. aureus* colonization and other respiratory viruses complicates the understanding of potential interactions between these pathogens.

## Concluding remarks

While clinical and mechanistic aspects of the synergism between *S. aureus* and influenza virus have been deeply studied, the interactions between this bacterium and other common respiratory viruses were only partially investigated [10]. Literature still lacks more data about the relations between *S. aureus* carriage and non-influenza respiratory virus infections, as well as deeper insights into mechanisms of interactions between these different pathogens. The evaluation of the concrete risk of switch from commensal *S. aureus* colonization to invasive disease during viral respiratory tract infections may help us to propose efficient strategies of decolonization when needed. Furthermore, the comprehension of the involved molecular pathways may help to develop new strategies of preventive and curative treatments.

CDHR3: Cadherin-Related Family Member 3; cFn: cellular Fibronectin; dsRNA: double-stranded RNA; ENA78: Epithelial Neutrophil Activating peptide-78; FnBP: Fibronectin Binding Protein; HRV: Human Rhinovirus; ICAM-1: InterCellular Adhesion Molecule 1; IFN: Interferon; IL: Interleukin; IRFs: Interferon Regulator Factors; IκB: NFκB Inhibitor; LDLR: Low-Density Lipoprotein Receptor; MDA5: Melanoma Differentiation Associated protein-5; NFκB: Nuclear Factor kappa-light-chain-enhancer of activated B cells; NKG2D: Natural Killer Group 2D receptor; Nod2: Nucleotide-binding oligomerization domain-containing protein 2; NOX: membrane-bound NADPH OXidase; Rac1: Ras-related C3 botulinum toxin substrate 1; RANTES: Regulated on Activation, Normal T cell Expressed and Secreted chemokine; RIG-I: Retinoic acid Inducible Gene-1; ROS: Reactive Oxygen Species; S. aureus: Staphylococcus aureus; SEA: Staphylococcal Enterotoxin A; SEB: Staphylococcal Enterotoxin B; ssRNA: single-stranded RNA; TLR: Toll-Like receptor; ZO-1: Zona Occludens 1.
